# Differential expression of transforming growth factor-β isoforms in bullous keratopathy corneas

**Published:** 2010-02-05

**Authors:** Barbara Strzalka-Mrozik, Agnieszka Stanik-Walentek, Malgorzata Kapral, Malgorzata Kowalczyk, Jolanta Adamska, Joanna Gola, Urszula Mazurek

**Affiliations:** 1 Department of Molecular Biology, Medical University of Silesia, Sosnowiec, Poland; 2 Department of Ophthalmology, Medical University of Silesia, Sosnowiec, Poland; 3 Department of Biochemistry, Medical University of Silesia, Sosnowiec, Poland; 4 Department of Medical Genetics, Medical University of Silesia, Sosnowiec, Poland

## Abstract

**Purpose:**

The aim of this study was to investigate transcriptional activities of genes encoding transforming growth factor (TGF)-β isoforms in bullous keratopathy corneas.

**Methods:**

The study group consisted of 45 patients with bullous keratopathy (22 females and 23 males). The control group included 45 corneal donors (21 females and 24 males). Quantification of *TGF-β1*, *TGF-β2*, and *TGF-β3* mRNAs was performed by real-time quantitative reverse transcription PCR (QRT-PCR).

**Results:**

*TGF-β1*, *TGF-β2*, and *TGF-β3* mRNAs were detected in both normal and pseudophakic bullous keratopathy (PBK) corneas. We found significantly lower transcriptional activity of *TGF-β3* mRNA in bullous keratopathy corneas compared to normal tissues. *TGF-β1* and *TGF-β2* expressions were at the same level in both PBK and healthy corneas.

**Conclusions:**

Downregulation of *TGF-β3* gene expression may play a significant role in molecular changes observed in bullous keratopathy.

## Introduction

Pseudophakic bullous keratopathy (PBK) is a complication of cataract surgery with intraocular lens placement and is an indication for corneal transplantation. Clinical hallmarks of this disease are chronic corneal edema due to corneal endothelial cell dysfunction, subepithelial bullae (blisters), and loss of transparency [1-3]. This disease is also characterized by extensive fibrosis with abnormal deposition of extracellular matrix proteins, tenascin-C, and fibrillin [1,4,5]. Moreover, PBK is often accompanied by scarring and neovascularization [3].

Various cytokines and growth factors are strongly involved in these processes [6,7]. One of the most important mediators is the family of transforming growth factors β (TGF-β), composed of five isoforms (TGF-β1-5) [8,9]. Among them, only TGF-β1, β2, and β3 are found in humans [9,10]. The TGF-β family of cytokines regulates such fundamental aspects of cellular function as cell growth, differentiation, inflammation, and wound healing [11-13]. In addition, there is substantial evidence suggesting participation of TGF-β in many human diseases [13-15], including fibrotic pathologies of the eye [16-18].

In vitro TGF-β isoforms have a similar effect on biologic tissues; however, in vivo they are generally characterized by varied degrees of expression and different functions. Their biologic activity depends on quantitative relationships between individual isoforms [19-21]. TGF-β1 and TGF-β2 isoforms have been reported to play a profibrotic role, whereas TGF-β3 possesses antifibrotic activity [22]. Embryonic wounds with a high level of TGF-β3 and low levels of TGF-β1 and TGF-β2 heal with no scarring [23]. During scar-forming in adults, however, TGF-β1 and TGF-β2 expression is significantly higher than TGF-β3 expression during wound healing. Such relationships during development of bullous keratopathy as a result of cornea injury after cataract surgery remain unclear.

Therefore, the present study focuses on transcriptional activities of genes encoding TGF-β1, TGF-β2, and TGF-β3 isoforms in human corneas with bullous keratopathy. Quantitative relationships between mRNA levels of these three isoforms were also assessed.

## Methods

### Tissues

Normal human corneas used as controls were taken within 12 h after death from 45 donors (21 females and 24 males; mean age 53.4 years; range 42–65 years). Inclusion criteria for becoming a corneal tissue donor were determined by the Eye Bank Association of America (EBAA).

The patient group involved 45 individuals (22 females and 23 males; mean age 56.1 years; range 45–65 years) with a clinical diagnosis of PBK, treated in the Department of Ophthalmology, Medical University of Silesia, St. Barbara Hospital, Katowice, Poland. The PBK diagnosis was based on the presence of chronic corneal stromal and epithelial edema, painful epithelial bullae with recurrent erosions as well as signs and symptoms of chronic ocular irritation. Exclusion criteria were as follows: the absence of inflammation and degeneration of anterior and posterior segment of eyeball, corneal neovascularization, diabetic retinopathy, pseudoexfoliation syndrome (PEX) and glaucoma. All patients were subjected to cataract surgery in the past; the difference in time between cataract surgery and corneal transplantation averaged 32.4 months. PBK corneas were obtained within 12 h of penetrating keratoplasty.

Surgical anesthesia was as follows: Fentanyl (2 mg), Midazolam (2 mg), Athropine (0,01 mg/kg body mass), Thiopental (4-5 mg/kg body mass), Vecuronium (0,1 mg/kg body mass). Because only the central corneal buttons (7.5 mm diameter) were available for PBK corneas, normal corneas were trephined, and only the central portions were used. Tissue specimens were stored in EUSOL C (Alchimia, Padova, Italy) at –70 °C for 24 h until RNA extraction. The research was approved by the Bioethics Committee of Medical University of Silesia, Katowice, Poland (NN-6501–146/06). All patients were informed about the research and signed an informed consent form.

### RNA extraction from tissue specimens

Total RNA was extracted from the specimens using a commercially available kit (Total RNA Prep Plus Kit; A&A Biotechnology, Gdansk, Poland) based on acid guanidinium-thiocyanate phenol-chloroform method by Chomczynski and Sacchi, according to the manufacturer's instructions. RNA extracts were treated with DNase I (MBI Fermentas, Vilnius, Lithuania). The quality of extracts was checked electrophoretically using an 0.8% agarose (Sigma-Aldrich, Munich, Germany) gel stained with ethidium bromide (Sigma-Aldrich). Results were analyzed and recorded using the gel documentation system 1D Bas-Sys (Biotech-Fisher, Perth, Australia). Total RNA concentration was determined by spectrophotometric measurement using the Gene Quant II RNA/DNA Calculator (Pharmacia Biotech, Cambridge, UK).

### Real-time quantitative reverse transcription-PCR assay

Transcriptional activities of *TGF-β1*, *TGF-β2*, *TGF-β3*, and glyceraldehyde-3-phosphate dehydrogenase (*GAPDH*) genes were evaluated using real time quantitative reverse transcription (QRT)-PCR and SYBR Green I chemistry (QuantiTect® SYBR® Green RT-PCR kit; QIAGEN, Valencia, CA). Analysis was performed using an Opticon™ DNA Engine Continuous Fluorescence Detector (MJ Research, Watertown, MA). All samples were tested in triplicate. *GAPDH* was included to monitor the QRT-PCR efficiency. Oligonucleotide primers specific for *TGF-β1*, *TGF-β2*, *TGF-β3*, and *GAPDH* genes were described previously by Strzalka et al. [24,25] and Ercolani et al. [26], respectively (Table 1). The thermal profile for one-step RT-PCR was as follows: reverse transcription at 50 °C for 30 min, denaturation at 95 °C for 15 min, 50 cycles consisting of temperatures 94 °C for 15 s, 60 °C for 30 s, and 72 °C for 30 s. To detect the expression profile of each investigated gene, commercially available standards of β-actin (*ACTB*) cDNA (TaqMan^®^ DNA Template Reagent kit; PE Applied Biosystems, Inc., Foster, CA) were used at five different concentrations (ranging from 400 to 8,000 copies of *ACTB* cDNA), as recommended by Bustin [27]. Amplification plots for each standard template were used to determine the cycle threshold values (Ct). A standard curve was generated by plotting the Ct values against the log of the known amount of the *ACTB* cDNA copy number. The obtained results of the mRNA copy number were recalculated per 1 μg of total RNA. Each run was completed using melting curve analysis to confirm specificity of the amplification and absence of the primer dimers. The RT-PCR products were also separated in 6% polyacrylamide gels (PAA) and visualized with silver salts.

**Table 1:** Characteristic of primers used for amplification.

**Gene**	**Sequence of primers**	**Length of amplicon (bp)**	**Tm (ºC)**
*GAPDH*	Forward: 5’-GAAGGTGAAGGTCGGAGTC-3’	226	80
	Reverse: 5’-GAAGATGGTGATGGGATTC-3’		
*TGFβ-1*	Forward:5’TGAACCGGCCTTTCCTGCTTCTCATG3’	151	85
	Reverse: 5’GCGGAAGTCAATGTACAGCTGCCGC3’		
*TGFβ-2*	Forward: 5’TACTACGCCAAGGAGGTTTACAAA3’	201	80
	Reverse: 5’TTGTTCAGGCACTCTGGCTTT3’		
*TGFβ-3*	Forward: 5’CTGGATTGTGGTTCCATGCA3’	121	81
	Reverse: 5’TCCCCGAATGCCTCACAT3’		

In the table, bp indicates base pairs and Tm indicates melting temperature.

### Statistical analyses

Statistical analyses were performed using Statistica 8.0 software (StatSoft, Tulsa, OK), with a significance level set at p<0.05. Values are expressed as median (Me), minimum, and maximum. The Kruskal–Wallis one-way analysis of variance test and post hoc multiple test based on the average ranks were applied to assess differences in the expression of *TGF-β* isoforms in normal and pathological tissues. Comparison of transcriptional activity of examined genes between normal and PBK corneas was made using the Mann–Whitney *U* test.

## Results

**Figure 1:**
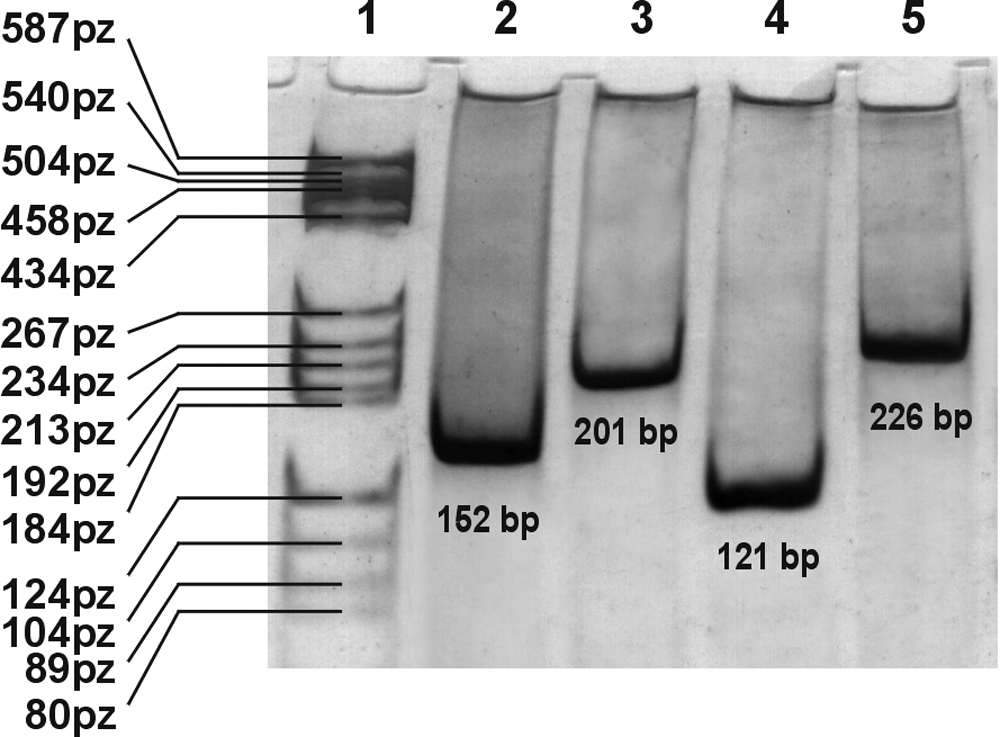
Reverse transcription PCR products separated in 6% polyacrylamide gel. lane 1, marker of size pBR 322/BsuRI (MBI Fermentas, Vilnius, Lithuania); lane 2, transforming growth factor -β1 (152 base pair, bp); lane 3, transforming growth factor -β2 (201 bp); lane 4, transforming growth factor -β3  (121 bp); lane 5 glyceraldehyde-3-phosphate dehydrogenase (226 bp).

In the present study, transcriptional activity of *TGF-β* isoforms in both normal and bullous keratopathy human corneas was determined using real-time QRT-PCR. In the first step of the study, specificity of the RT-PCR assay for the target genes was confirmed experimentally on the basis of the amplimers’ melting temperatures. For each RT-PCR product, a single peak at the expected temperature was observed: *TGF-β1* 85.4 °C; *TGF-β2* 80.0 °C; *TGF-β3* 80.6 °C; *GAPDH* 80.1 °C (data not shown). Gel electrophoresis also revealed the presence of a single product of the predicted length (Figure 1).

**Figure 2:**
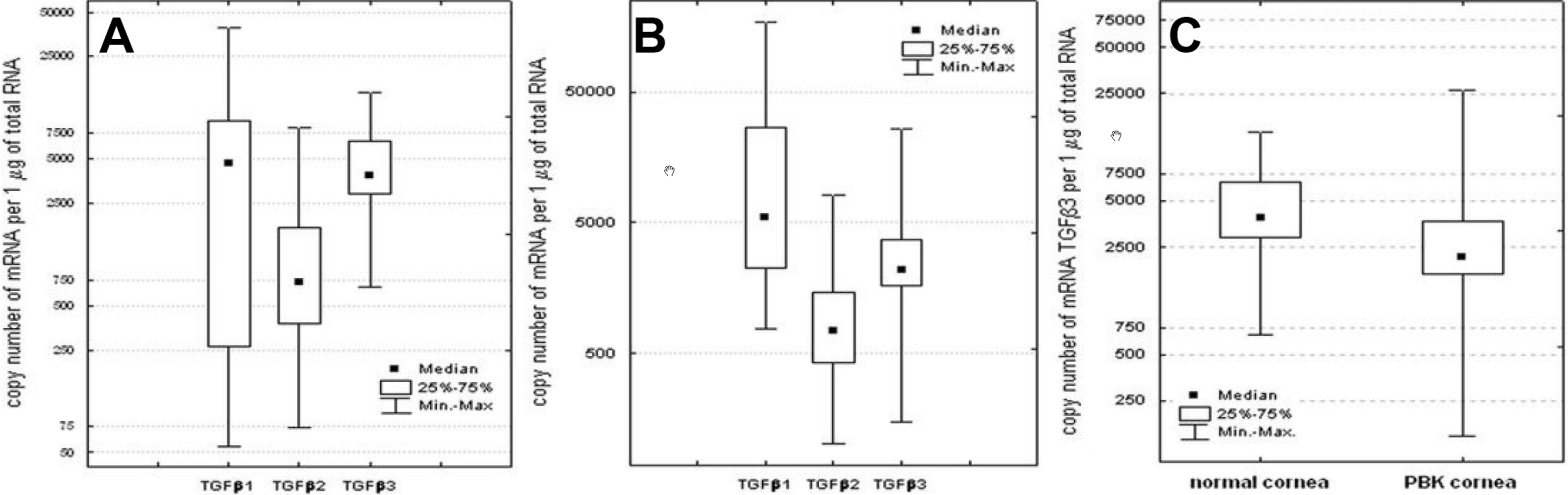
Transforming growth factor β in normal human corneas and pseudophakic bullous keratopathy corneas. The expression of  transforming growth factor -β1, transforming growth factor -β2, and transforming growth factor -β3 isoforms in (**A**) normal human corneas (Kruskal–Wallis one-way analysis of variance test; p=0.0003) and (**B**) pseudophakic bullous keratopathy corneas (Kruskal–Wallis one-way analysis of variance test; p<0.0001). **C**: Comparison of transforming growth factor -β3 gene expression between pseudophakic bullous keratopathy and normal corneas (Mann–Whitney U test, p=0.0107).

In the next step, levels of *TGF-β1*, *TGF-β2*, and *TGF-β3* mRNAs in normal and bullous keratopathy human corneas were assessed and the quantitative relations among the mRNA of these three isoforms were then evaluated (Figure 2A,B). *TGF-β1*, *TGF-β2*, and *TGF-β3* isoforms were detected in all tested samples obtained from normal corneas (*TGF-β1* Me=4,693.0 copies/μg RNA; *TGF-β2* Me=719.0 copies/μg RNA; *TGF-β3* Me=3,844.7 copies/μg RNA) and bullous keratopathy corneas (*TGF-β1* Me=5,553.0 copies/μg RNA; *TGF-β2* Me=738.9 copies/μg RNA; *TGF-β3* Me=2,176.5 copies/μg RNA). Comparable analysis of all *TGF-β* mRNA copies/μg of total RNA revealed the following relationships in healthy cornea: *TGF-β1*>*TGF-β2* (p=0.0164, post hoc test); *TGF-β3*>*TGF-β2* (p<0.001, post hoc test); *TGF-β1*=*TGF-β3* (not significant [NS], post hoc test). Pathologically changed cornea relationships were similar to that observed in normal cornea: *TGF-β1*>*TGF-β2* (p<0.001, post hoc test); *TGF-β3*>*TGF-β2* (p=0.0221, post hoc test); *TGF-β1*=*TGF-β3* (NS, post hoc test). In PBK corneas *TGF-β3* mRNA expression was found to be significantly lower (Mann–Whitney *U* test, p=0.0107) compared to normal tissues (Figure 2C). However, transcriptional activity of the *TGF-β1* (p=0.0585) and *TGF-β2* (p=0.5540) genes in both healthy and PBK corneas was at the same level.

## Discussion

The role of *TGF-β1*, *TGF-β2*, and *TGF-β3* in the cornea is relatively well understood [10,13,28]. However, quantitative relationships between mRNA expressions of different isoforms in the course of some corneal pathologies are still unclear. In previously published reports mRNA expression of *TGF-β* was evaluated mostly in healthy tissues [10,21,24,25], and only a few authors have analyzed the expression profile of TGF-β1, TGF-β2, and TGF-β3 in the course of bullous keratopathy [1,3,5].

In the present study real-time RT-PCR was used to examine the mRNA expression of genes encoding *TGF-β* isoforms in human normal and pathologically changed cornea. Transcriptional activity was measured on the basis of the mRNA copy number per 1 μg of total RNA, following the recommendations of Tricarico et al. [29]. Transcripts of all three *TGF-β* isoforms were detected in PBK corneas and in healthy ones, which is in agreement with other published results when the examined material constituted cell cultures [10,30] or rat corneal epithelium [13,28].

Li et al. [21] reported that *TGF-β1* transcriptional activity was the highest in all tested parts of the anatomy of the eye. However, they studied the expression of genes encoding isoforms of *TGF-β* only in the healthy human cornea. This remains partly consistent with current results showing *TGF-β1* and *TGF-β3* as the predominant isoforms both in human healthy cornea and in affected cornea. Similar results were also demonstrated by Carrington et al. [31] and Tseng et al. [32]. Carrington et al. found *TGF-β1* to be the predominant isoform in the bovine cornea during wound healing. Tseng et al. postulated that healthy human cornea is characterized by high transcriptional activity of *TGF-β1*. However, Tuli et al. [12], based on investigations using animal models, revealed that damage of the corneal surface leads to an increase in expression of genes encoding *TGF-β2* and *TGF-β3*.

Of importance here is that only a fraction of previous studies shows quantitative relationships between *TGF-β* isoforms in the course of bullous keratopathy. Saghizadeh et al. [33] evaluated expression of the *TGF-β2* isoform both at the mRNA and protein levels in PBK and normal cornea; however statistically significant differences were not found. Kenney et al. [2] performed similar studies but revealed a significant increase in transcriptional activity of genes encoding isoforms of *TGF-β1* and *TGF-β2* in the course of bullous keratopathy. Their report contradicts our findings, which demonstrated that the differences in mRNA expression of both *TGF-β1* and *TGF-β2* genes in patients with bullous keratopathy compared to the control group were not statistically significant.

Interestingly, transcriptional activity of TGF-β3 was reduced in PBK compared to the control group. Data are lacking regarding *TGF-β3* expression in bullous keratopathy. Downregulation of transcriptional activity of *TGF-β3* in the present study may have been caused by the loss of keratinocytes observed in the course of PBK [34]. On the other hand, molecular mechanisms leading to a decrease in the *TGF-β3* mRNA level cannot be ruled out. After cataract surgery epithelial cells undergo epithelial-mesenchymal transition (EMT) [35]. In this process not only the morphology but also the transcriptional program of the epithelial cells is altered. After epithelial-mesenchymal transition cells become capable of expressing components of the extracellular matrix and probably other molecules, which can lead to reduced *TGF-β3* gene expression. The *TGF-β3* isoform is a potential therapeutic agent of corneal repair, especially as it has no harmful effect on corneal re-epithelialization [31]. Thus, early application of TGF-β3 during or shortly after cataract surgery would prevent patients from such complications as PBK. The question remains about whether such treatment in patients with bullous kerathopathy could restore normal corneal morphology, taking into account the role of TGF-β3 in tissue remodeling after wounding [22].

Summarizing the results of the present study, all three isoforms were found to be differentially expressed in the course of bullous kerathopathy, but only *TGF-β3* was changed compared to normal cornea. Obtained data suggest that decreased expression of *TGF-β3* may play a significant role in molecular changes observed in bullous keratopathy.
